# Supplementary material for: The Brown Alga Bifurcaria bifurcata Presents an Anthelmintic Activity on All Developmental Stages of the Parasitic Nematode Heligmosomoides polygyrus bakeri
Source: Pathogens. 2023 Mar 30;12(4):540. doi: 10.3390/pathogens12040540 (PMC10144559; doi:10.3390/pathogens12040540)
Supplement: Supplementary file 1 [file pathogens-12-00540-s001.zip › pathogens-2266509-supplementary.pdf]

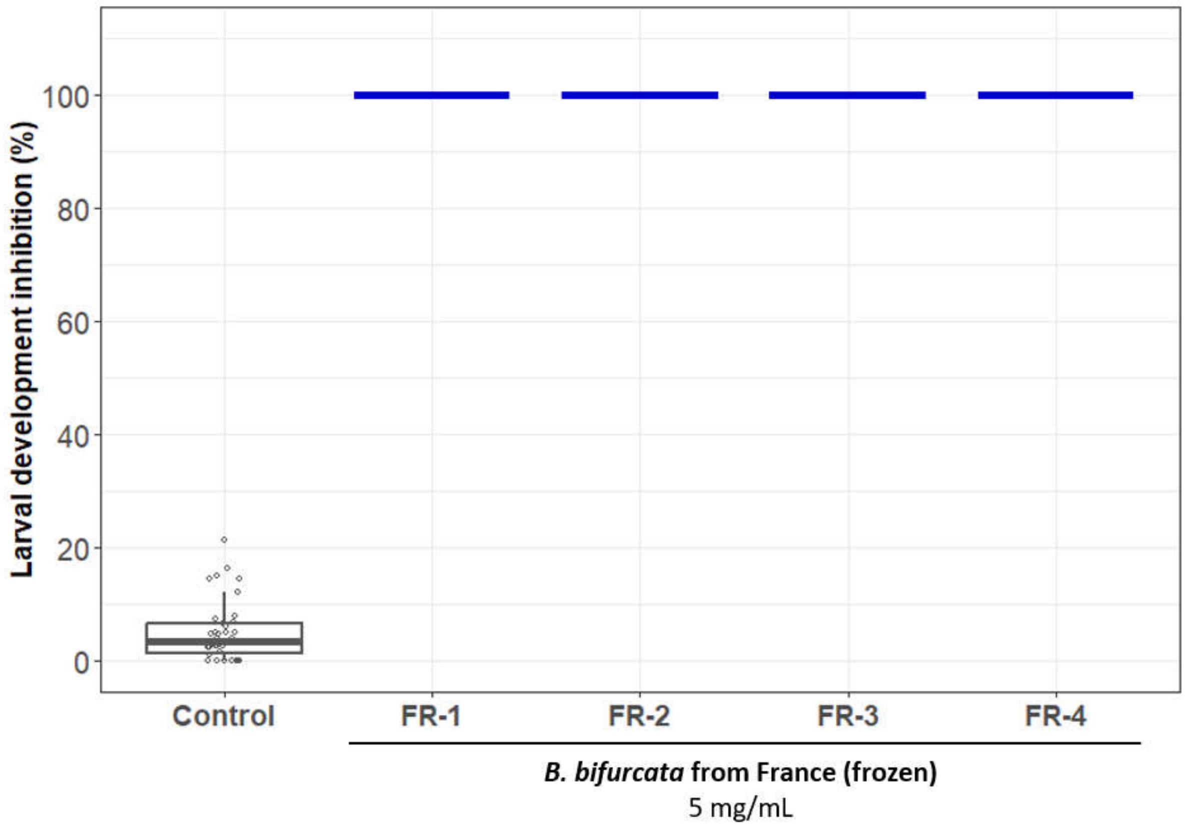

Figure S1. Comparison of different batches of *B. bifurcata* from France (frozen) for the inhibition of the larval development of *H. polygyrus bakeri*. Percentage inhibition of development of *H. polygyrus bakeri*, from L1-L2 to L3, in presence of water (control), or different batches of *B. bifurcata* at 5 mg/mL. The algae from France were collected in 2019 (FR-1) and 2021 (FR-2, FR-3, FR-4) and were frozen either directly (FR-2), after 24 h at + 4 °C (FR-3), or after 48 h at + 4 °C (FR-1 and FR-3). Results are from at least three independent experiments, with *n* = 6 per sample each time.

Table S1. Mass evaluation of the different organic extractions performed on the two algal batches of *B. bifurcata*, frozen from France and dried from Portugal. These results are obtained from 2 g of freeze-dried aqueous extract.

	**Solvent use**	**Algae frozen from France**	**Algae dried from Portugal**
Mean of the mass after extraction (mg) (percentage of initial biomass (%))	Heptane	9.33 mg (0.47%)	10.13 mg (0.51%)
	EtOAc	32.10 mg (1.61%)	47.9 mg (2.40%)
	BuOH	61.70 mg (3.09%)	52.47 mg (2.62%)

Results are from three independent experiments, with *n* = 6 per sample each time.
